# Cxbladder Triage in Haematuria Assessment Reduces Time From Referral to Specialist Assessment and Bladder Cancer Treatment and Improves Equity for Māori

**DOI:** 10.1111/ans.70960

**Published:** 2026-09-07

**Authors:** Daniel Eaton, Melissa Kerdemelidis, Giovanni Losco, Stephen Mark, Maira Patu, Nikki Elliot, Chris Frampton

**Affiliations:** ^1^ Department of Urology Health NZ Waitaha Canterbury Christchurch New Zealand; ^2^ Population Health, Planning, Funding & Outcomes Health NZ Waitaha Canterbury Christchurch New Zealand; ^3^ Department of Surgery University of Otago Christchurch Christchurch New Zealand; ^4^ Maori Indiginous Health Innovation University of Otago Christchurch Christchurch New Zealand; ^5^ Department of Radiology Health NZ Waitaha Canterbury Christchurch New Zealand; ^6^ Department of Medicine University of Otago Christchurch Christchurch New Zealand

**Keywords:** bladder cancer, haematuria, health equity, pharmacological biomarkers, transitional call carcinoma

## Abstract

**Background:**

Bladder cancer is responsible for considerable morbidity and mortality. Patients commonly present with haematuria to primary care for assessment. In Canterbury, New Zealand, the first‐line investigations available to primary care were changed in 2018, from urine cytology to CxBladder Triage (CxBT) and upper urinary tract imaging. This retrospective study was performed to evaluate the impact of this change in diagnostics.

**Methods:**

A retrospective audit of routinely collected electronic data from March 2013 to December 2021 from Christchurch Hospital, New Zealand, compared the traditional primary care haematuria pathway with the CxBT haematuria pathway. The primary outcome was waiting time from primary care referral to urological specialist appointment and waiting time to first operative treatment for bladder cancer. Waiting time was further analysed for ethnicity. The secondary outcome was monthly haematuria referrals for urological specialist appointment.

**Results:**

There were 4512 haematuria referrals during the study period. Following the introduction of CxBT to the primary care haematuria pathway, waiting time from primary care referral to urological specialist review reduced by 14.8 days and time to surgery by 15.0 days. There was a greater reduction for Māori compared with that of non‐Māori patients. The monthly haematuria referrals for urological specialist appointment reduced on average by 6.9 referrals per month.

**Conclusion:**

CxBT in primary care haematuria pathway reduces waiting time from primary care referral to urological specialist appointment and to first operative treatment for bladder cancer, whilst also reducing monthly referrals for urological specialist appointments and improving equity for Māori.

## 1 Introduction

Bladder cancer is the tenth most common cancer worldwide and accounts for considerable morbidity and mortality [1]. Many patients present to primary care providers with haematuria and are then referred for a urological specialist appointment. The referral criteria and guidelines vary globally. The principal aim is to rule out urological malignancy [2, 3, 4]. In Christchurch, New Zealand, primary care providers access local haematuria guidelines via a local website Community HealthPathways [4]. This provides guidance for community‐based investigations and the criteria for publicly funded specialist referral. In primary care, prior to January 2018, patients with haematuria were assessed by urine cytology, urine microscopy, culture and sensitivities and upper urinary tract radiology (either ultrasound or computed tomography depending on age and presence of macroscopic or microscopic haematuria). If criteria were met, patients were then referred for a publicly funded urological specialist appointment, where a cystoscopy would usually be performed.

In recent years, novel assays which detect prognostic urinary biomarkers for bladder cancer have become available [5, 6]. One such assay is a urine test called Cxbladder Triage (CxBT) (Pacific Edge Limited, Centre for Innovation, 87 St David Street, Dunedin, New Zealand) which detects the expression of five urinary mRNA biomarkers (CDC2, MDK, IGFBP5, HOXA‐13 and CXCR2) which may be present in the urine of patients with bladder cancer [7]. A CxBT score can be generated using an algorithm which includes these five urinary biomarkers and takes into account four phenotypic factors: age, sex, smoking status and haematuria frequency. A CxBT score of 4.0 or greater was considered positive, mandating a referral for a urological specialist appointment. A CxBT score less than 4.0 was regarded as negative, with a very low probability of bladder cancer. The clinical utility of CxBT has been evaluated in several studies at Christchurch Hospital, New Zealand [8, 9]. The combination of a negative CxBT score and normal upper tract imaging showed an impressive negative predictive value of 99.8% for bladder cancer [8]. This finding was again validated in a further study which showed a negative CxBT and urinary tract imaging had a negative predictive value of 99.9% for detecting bladder cancer, with 98.1% sensitivity and 99.9% specificity [9]. These results prompted a Canterbury clinical working group of both primary care and Urology services to update the local haematuria guidelines, removing urine cytology and introducing CxBT. CxBT was requested in primary care, by the patients' general practitioner. The result was interpreted by primary care. Given the extremely low risk of urological malignancy in haematuria patients with a negative CxBT score and normal upper tract imaging, referral for urological specialist appointment and cystoscopy was considered to no longer be required. However, if macroscopic haematuria persisted for more than 3 months, the community‐based investigations were then repeated [4].

A previous pilot study evaluated a smaller haematuria dataset in 2020, and found some potential inequities in waiting times, which warranted further investigation over a longer time frame and on a larger dataset. As part of this work, Canterbury Urology identified haematuria as a high‐volume, serious condition, which could be analysed for Māori health inequities.

This retrospective study was performed to evaluate the impact of this change of test on waiting times from primary care referral to urological specialist appointment, and waiting time to first operative treatment for bladder cancer. Any impacts on the number of haematuria referrals for urological specialist appointment, and impacts on Māori health equity were also considered.

## 2 Methods

### 2.1 Study Design

A retrospective audit of all haematuria patients referred from primary care for a urological specialist appointment at Christchurch Hospital, a publicly‐funded facility, between March 2013 and December 2021 was performed. Patients were divided into two groups depending on the time of referral: pre‐CxBT group included referrals from March 2013 to December 2017 while the post‐CxBT group included referrals from January 2018 to December 2021. The primary outcomes were waiting time from primary care referral to urological specialist appointment and waiting time to first operative treatment for bladder cancer. Waiting time was further analysed for any ethnicity‐related differences or avoidable health inequalities. The third outcome considered was any impacts on monthly haematuria referrals for urological specialist appointments.

### 2.2 Participants and Data Collection

Haematuria patients were identified from two electronic sources. Firstly, referral records to Christchurch Hospital for haematuria were collected and then confirmed with patients registered on the Christchurch Hospital haematuria database. Demographic data including age, ethnicity and gender were recorded. Diagnostic data including urine cytology (for earlier patients), CxBT and upper tract imaging were retrieved. Other data included date of referral, date of urological specialist appointment and date of first operative treatment for bladder cancer, where required.

### 2.3 Statistical Analysis

The waiting times are summarised as medians and interquartile ranges and compared between groups using non‐parametric Mann–Whitney U tests. All analyses were undertaken using SPSS v30.0 and a two‐tailed *p* value < 0.05 was defined as indicating statistical significance.

### 2.4 Ethical Approval and Patient Consent

As this was a retrospective audit of unidentifiable patients, with no patient contact, this did not require Health and Disability Ethics Approval review and was formally considered out of scope (11 January 2022). Locality approval with project registration was obtained from the Research Office at Health New Zealand Te Whatu Ora Waitaha Canterbury (RO# 22162) on 20 July 2022. Consultation and formal approval from the Christchurch Hospital Māori Research Advisor was granted on 11 January 2023.

## 3 Results

A total of 4512 haematuria referrals were received in the study period (Table 1). The majority of referrals were from males (*n* = 3227, 71.5%). Europeans were the largest ethnic group (*n* = 3884, 86.1%), followed by Māori (*n* = 282, 6.3%), Asian (*n* = 183, 4.1%) and Pacific Peoples (*n* = 72, 1.6%). The mean age of the study cohort was 65.3 years, however when analysed by ethnicity, Māori, Pacific Peoples and Asian patients had much younger mean ages of 55.2, 55.8 and 53.9 years respectively compared to that of Europeans (66.9 years).

**TABLE 1:** Patient demographics.

	Ethnicity		Gender		Age
	Ethnicity	Total (%)	Female (%)	Male (%)	Mean (yr)
Combined	European	3884 (86.1)	1054 (27.1)	2830 (72.9)	66.9
	Māori	282 (6.3)	102 (36.2)	180 (63.8)	55.2
	Asian	183 (4.1)	61 (33.3)	122 (66.7)	53.9
	Pacific	72 (1.6)	28 (38.9)	44 (61.1)	55.8
	Other ethnicities	91 (2.0)	40 (44.0)	51 (56.0)	56.9
	Total	4512	1285 (28.5)	3227 (71.5)	65.3
Pre‐CxBT	European	2266 (85.6)	656 (28.9)	1610 (71.1)	
	Māori	174 (6.6)	67 (38.5)	107 (61.5)	
	Asian	103 (3.9)	39 (37.9)	64 (62.1)	
	Pacific	54 (2.0)	23 (42.6)	31 (57.4)	
	Other ethnicities	51 (1.9)	26 (51.0)	25 (49.0)	
	Total	2648	811	1837	
Post‐CxBT	European	1618 (86.8)	398 (24.6)	1220 (75.4)	
	Māori	108 (5.8)	35 (32.4)	73 (67.6)	
	Asian	80 (4.3)	22 (27.5)	58 (72.5)	
	Pacific	18 (1.0)	5 (27.8)	13 (72.2)	
	Other ethnicities	40 (2.1)	14 (35.0)	26 (65.0)	
	Total	1864	474	1390	

There was a significant reduction of 14.8 days (*p* < 0.001) in the overall median waiting time from primary care referral to urological specialist appointment in the post‐CxBT group, compared to the pre‐CxBT group (43.0 vs. 57.8 days respectively) (Table 2). When analysed by ethnicity there was a statistically significant greater reduction in median waiting time from primary care referral to urological specialist appointment for Māori patients compared with that in European patients (14.4 vs. 13.8 days, *p* < 0.001).

**TABLE 2:** Median waiting time from primary care referral to urological specialist appointment, grouped by ethnicity.

	Total	European	Māori	Asian	Pacific	Other ethnicities
Pre‐CxBT group						
Median (days)	57.8	56.6	58.4	58.6	56.8	57.6
Post‐CxBT group						
Median (days)	43.0	42.6	43.5	48.0	50.6	51.0
Change in wait time from pre‐CxBT compared with post‐CxBT group						
Median (days)	−14.8 (*p* < 0.001)	−13.8 (*p* < 0.001)	−14.4 (*p* < 0.001)	−11.0 (*p* = 0.09)	−5.0 (*p* = 0.68)	−5.1 (*p* = 0.992)

There was a reduction of 15.0 days (*p* < 0.001) in the overall median waiting time from primary care referral to first operative treatment of bladder cancer in the post‐CxBT group compared to the pre‐CxBT group (63 vs. 78 days respectively) (Table 3). Similar to the waiting time for primary care referral to urological specialist appointment, when analysed by ethnicity, there was a greater reduction in median waiting time from primary care referral to first operative treatment for Māori patients compared to European patients (20.0 days vs. 16.0 days, respectively).

**TABLE 3:** Median waiting time from primary care referral to first operative treatment for bladder cancer, grouped by ethnicity.

	Total	European	Māori	Asian	Pacific	Other ethnicities
Pre‐CxBT group						
Median (days)	78.0	78.0	90.0	74.0	78.0	51.5
Post‐CxBT group						
Median (days)	63.0	62.0	70.0	58.0	78.0	73.5
Change in wait time from pre‐CxBT compared with post‐CxBT group						
Median (days)	−15.0 (*p* < 0.001)	−16.0 (*p* < 0.001)	−20.0 (*p* = 0.355)	−16.0 (*p* = 0.380)	0	22.0 (*p* = 0.064)

There was an overall reduction in the number of monthly haematuria referrals for urological specialist appointment in the post‐CxBT group compared with the pre‐CxBT group (Table 4). In total, 2648 referrals were received in the pre‐CxBT period (March 2013 to December 2017), while 1864 referrals were received in the post‐CxBT period (January 2018 to December 2021). When averaged monthly, this showed a reduction of 6.9 referrals per month (45.7 vs. 38.8 referrals per month, respectively).

**TABLE 4:** Total and mean haematuria referrals in pre‐CxBT and post‐CxBT groups.

Groups	Months	Total referrals	Referrals per year	Referrals per month
Pre‐CxBT	58	2648	548	45.7
Post‐CxBT	48	1864	466	38.8

## 4 Discussion

### 4.1 Key Results

These results demonstrate that the addition of CxBT into the local primary care haematuria pathway had a significant and positive impact for the urology service. Waiting time from primary care referral to urological specialist appointment for all patients was reduced by over 2 weeks. Waiting times to first operative treatment of bladder cancer also reduced. Waiting time reductions are critically important as they lead to earlier diagnosis and treatment of time‐sensitive cancers. Of all the urological cancers, bladder cancer is one of the more rapidly progressive with early investigation and treatments known to save lives [1, 10]. Furthermore, reduction in waiting times is well recognised to have a psychological benefit for patients [11]. Additional benefits include a 15% reduction in average monthly haematuria referrals for urological specialist appointment, 6.9 per month or 82.8 per year. This is particularly important as unnecessary and costly urological specialist appointments and cystoscopies are avoided, allowing redirection of limited resources to other urological conditions, thereby allowing for more timely review of patients. It is likely that this reduction in referrals is a significant factor in the reduction in waiting time for urological specialist appointment. Cystoscopy itself also carries morbidity, such as urinary tract infection, sepsis, urinary retention and exacerbation of haematuria. Avoiding cystoscopy therefore further benefits both the patient and the health system.

The introduction of CxBT assisted primary care to take the lead in the care of patients with haematuria. Patients with negative investigations did not require specialist referral, allowing them to have their care based entirely in the community. For these patients, this would allow significant psychological benefit and reduced health‐related anxiety by early reassurance of benign haematuria. Community‐based screening such as CxBT is also known to be more acceptable to Maori, such as the recent introduction of self‐administered testing for haematuria [12]. Other benefits include financial savings with reduced travel costs and time off work for urological specialist appointments, although there may be an additional cost associated with primary care consultations as these are only partially publicly funded in the New Zealand setting. Whilst bladder cancer presentations and haematuria are uncommon in the primary care setting, consideration should also be given to the increased workload borne by primary care with this pathway.

### 4.2 Ethnicity and Improving Equity

Māori are the indigenous or First Nations population in New Zealand and according to the 2023 Census represent 17.8% of the population and 10.6% of the population in the Canterbury region [13]. Māori health inequities in cancer incidence and mortality are well documented by Te Aho o Te Kahu (Cancer Control Agency), showing a substantial gap in survival rates between Māori and non‐Māori for the most common cancers, including bladder cancer [13]. Of concern, Māori with bladder cancer had approximately 37% greater mortality compared to that of non‐Māori between 2007 and 2016. Our study noted a demographic difference between Māori and European patients with haematuria, with Māori being on average 10 years younger at the time of referral.

Ethnicity has been considered to be an evidence‐based marker of need in the New Zealand context, due to ethnic differences across a number of factors including exposure to risk factors, access to care and life expectancy [14, 15]. The underlying causes of determinants of ethnic health inequities in the New Zealand context have been hypothesized by Curtis et al. 2023, in their Te Kupenga Hauora Māori modified ‘Williams model’ for explaining indigenous and ethnic determinants of health [16, 17]. Further work has been done exploring ways to identify barriers and solutions to reduce areas of Māori health inequities in secondary care [18].

One of the health system factors driving disparities in cancer survival is access to early detection through evidence‐informed asymptomatic screening and prompt access through to secondary diagnostics [7, 10]. When analysed by ethnicity, our results showed a greater reduction in waiting time from primary care referral to urological specialist appointment in Māori patients compared to European patients. However, in keeping with these known disparities, Māori (and other ethnic minorities) still waited longer than European patients even after introduction of Cxbladder.

### 4.3 Other Areas to Investigate

A recent meta‐analysis of various urinary biomarkers argued against removing cystoscopy from diagnostic haematuria pathways [6]. However, both local studies by Davidson et al. [8, 9] demonstrate very high community capture of malignancy with no evidence of any missed bladder cancer in the patient group who did not receive flexible cystoscopy. The pathway states patients with haematuria that persists beyond 3 months have investigations repeated. To date no additional cases of bladder cancer have been identified. If resources permitted, these patients and their long‐term outcomes could be followed up.

### 4.4 Study Limitations

The most important limitation of this study is that the time from first symptoms and from first presentation to the general practitioner were not measured. Therefore this study is unable to answer if overall waiting time from initial patient presentation with haematuria to diagnosis and treatment of bladder cancer is reduced. It is possible that there are delays with waiting for completion of CxBT. However, it is assumed that by reducing the number of referrals requiring urological specialist appointment, overall access to specialist care is improved.

This study only captured data from patients who had the health literacy, financial ability and physical means to seek assistance for their haematuria symptoms in primary care. General practises had to have the capacity to see the patients. In most cases in New Zealand, for people aged 14 and over, there is a cost associated with accessing primary care. Costs may be lower or free if the patient has a Community Services Card (for those on low‐income) or is accessing a Very Low Cost Access primary care practise. This study therefore excludes those who were not able to access primary care. In New Zealand, a referral from primary care is required to access hospital specialist services.

This study does not capture any unmet need in urological care for haematuria. Unmet need is notoriously difficult to quantify, and patient self‐identified needs are not always aligned with clinician opinions. Self‐reported unmet health needs have been noted to be higher among some populations in New Zealand, with unmet need for general practitioner visits due to cost being higher among Māori, Pacific Peoples, those with disabilities and more socioeconomically deprived adults in a 2024–2025 National Health Survey [19].

Furthermore, a single centre retrospective audit carries some limitations. Our results depend on the reliability of retrospectively collected data, albeit mitigated by the use of electronically collected data. The patients were identified using two electronic sources, with the relevant data then extracted electronically. Whilst our results achieved significance in our local centre, further studies are required to confirm potential generalisability across New Zealand and other healthcare jurisdictions.

Data limitations include only allowing for male/female gender categories (reflecting data collection systems), and prioritised ethnicity was used (counting participants once in a single ethnic category, even if several had been identified in the clinical record) [20].

## 5 Conclusion

CxBT in the primary care haematuria pathway reduces waiting time from primary care referral to urological specialist appointment and to first operative treatment for bladder cancer, whilst also reducing monthly referrals for urological specialist appointments and improving equity in wait times for Māori.

## Author Contributions

**Daniel Eaton:** writing – original draft, writing – review and editing, data curation, formal analysis. **Melissa Kerdemelidis:** conceptualization, investigation, funding acquisition, writing – original draft, methodology, validation, visualization, writing – review and editing, project administration, data curation, supervision. **Giovanni Losco:** investigation, writing – original draft, methodology, validation, visualization, writing – review and editing, project administration, data curation, supervision. **Stephen Mark:** conceptualization, investigation, methodology, validation, visualization, supervision, writing – review and editing. **Maira Patu:** conceptualization, investigation, funding acquisition, writing – review and editing, methodology. **Nikki Elliot:** conceptualization, investigation, writing – review and editing, methodology. **Chris Frampton:** writing – review and editing, software, formal analysis, data curation, supervision, validation, visualization.

## Funding

This work was supported by the Ministry of Health.

## Disclosure

Funding for the biostatistical analysis was provided via a grant from the Ministry of Health. The Ministry of Health's Service Improvement Scheme provided funding to support a pilot project to work on the identification of barriers and solutions to reduce areas of Māori health inequities in secondary care [18].

## Data Availability

The data that support the findings of this study are available from the corresponding author upon reasonable request.
